# People are poorly equipped to detect AI-powered voice clones

**DOI:** 10.1038/s41598-025-94170-3

**Published:** 2025-03-31

**Authors:** Sarah Barrington, Emily A. Cooper, Hany Farid

**Affiliations:** 1 School of Information, University of California, Berkeley, CA 94720 USA; 2 Herbert Wertheim School of Optometry, University of California, Berkeley, CA 94720 USA; 3 Helen Wills Neuroscience Institute, University of California, Berkeley, CA 94720 USA; 4 Electrical Engineering and Computer Sciences, University of California, Berkeley, CA 94720 USA

**Keywords:** Information technology, Computer science

## Abstract

As generative artificial intelligence (AI) continues its ballistic trajectory, everything from text to audio, image, and video generation continues to improve at mimicking human-generated content. Through a series of perceptual studies, we report on the realism of AI-generated voices in terms of identity matching and naturalness. We find human participants cannot consistently identify recordings of AI-generated voices. Specifically, participants perceived the identity of an AI-generated voice to be the same as its real counterpart approximately $80\%$ of the time, and correctly identified a voice as AI generated only about $60\%$ of the time.

In January 2024, in the lead up to the November United States presidential election, an estimated tens of thousands of Democratic party voters received a robocall in the voice of President Biden instructing them not to vote in the upcoming New Hampshire primaries. The voice was AI-generated.

The perpetrators of this attempted election interference were Steven Kramer (a political consultant), Paul Carpenter (a magician and hypnotist who was paid $150 to create the fake audio), and a telecommunications company called Lingo Telecom^1,2^. Carpenter used ElevenLabs, a platform offering instant voice cloning for as little as $5 a month. Kramer was fined $6 million and subsequently charged with two dozen crimes including impersonating a candidate and voter suppression, while the telecommunications company, Lingo Telecom, received a $1 million fine for transmitting the calls. This is just one of many examples of how the rise of generative AI is being weaponized, from election interference, to disinformation campaigns^3^, to small-^4^ and large-scale^5^ financial fraud.

There is large literature on technologies that can automatically determine whether media – such as audio, video, and images – has been manipulated either by humans or generative AI^6^. These techniques, however, largely operate asynchronously and not as an audio or video call is unfolding in real time. The synchronous detection of fraudulent media, such as the phone calls that attempted to suppress voter turnout in New Hampshire, still poses significant technological challenges. Until technology can monitor every landline, mobile device, and video call (which itself would raise additional privacy concerns), people are largely left to their own defenses to sort out the real from the fake.

The question then naturally arises: how well-equipped are people for the perceptual challenge of distinguishing real from AI-generated content? The answer, of course, depends on both the quality of the fake and the modality of the media. For example, studies focusing on visual perception of images of people have concluded that participants are at chance at distinguishing real and AI-generated head shots^7,8^. Results for video (with audio) are more mixed – likely due to differences in the types of videos that have been assessed. While some studies report that performance is only slightly better than chance for videos of people talking^9,10^, a recent large-scale study investigating how well people could distinguish fabricated political speeches from real ones report a consistent accuracy of $80\%$ and above^11^.

Here, we focus on people’s ability to distinguish real voices from AI-generated voice clones, as would be required to detect a fraudulent phone call or voicemail. Interestingly, prior work suggests that people are better at this task than detection of AI-manipulated images, but can still often be tricked. For example, Mai et al.^12^ report that human participants were able to accurately distinguish real voices from AI-generated voice clones with an accuracy of $70\%$. This study, however, only used a single English and a single Chinese speaker identity, and the spoken phrases consisted of a single sentence ranging in length from 2 to 11 seconds (by comparison the fake Biden robocall was 40 seconds in length). Müller et al.^13^ report a similar accuracy of $80\%$. This second study has the advantage that it employed multiple speaker identities (107), but the spoken phrases were still relatively short at one to two sentences in length. Mostly consistent with these earlier studies, a recent study by Warren et al.^14^ found that human participants detect AI-generated voices with an accuracy of $73\%$. For each of these studies, the AI-generated voices were not created using state-of-the-art, commercially available techniques, and both studies focused on naturalness (is the voice real or not) and did not examine identity perception (who is speaking).

Groh et al.^11^ also investigated audio-only performance with political speeches. Performance when people were only given the isolated audio of the speeches was worse than with the full video with audio, but still above chance. Political speeches, however, are atypical given that the speakers are highly familiar to most listeners, and that political speeches have characteristic cadences and content that do not necessarily reflect natural conversational speech.

We expand on these previous studies by employing the state-of-the-art voice cloning of ElevenLabs (used in the Biden robocall), increasing the number of speakers to over 200, and considering how different tasks (identity and naturalness) impact our ability to distinguish AI-generated voices. This study reveals that, generally speaking, people are poorly equipped to identify AI-generated voice clones, both in terms of identity matching and naturalness.

## Methods

### Real speaker dataset

The stimuli for our study were sampled from the DeepSpeak dataset, which comprises recordings of 220 unique speakers collected through the Prolific research recruitment platform^15^. More details about this dataset can be found in the accompanying manuscript, but here we provide a brief summary.

All speakers were native English speakers and U.S. residents. Their ages ranged from 18-75 years (mean=38, sd=11.4), with 109 identifying as male, 107 female, and 4 non-binary. Racial identities included 158 White/Caucasian, 39 Black/African American, 26 Asian, 4 American Indian/Alaska Native, 2 Native Hawaiian/Other Pacific Islander, and 5 other.

Each speaker was instructed to record themselves responding to 32 prompts. The prompts were divided into four categories: (1) standardized scripted responses in which each speaker read the same prompt extracted from transcripts of the TIMIT dataset^16^; (2) randomized scripted responses in which each speaker read a randomized prompt from TIMIT; (3) unscripted responses in which each speaker responded to four open-ended questions, and asked for a response that was close to 30 seconds in length; and (4) combined responses consisting of four open-ended unscripted questions in which each speaker read out loud a question and then answered the question.

Both audio and video were recorded using a custom-built web application. The audio/video recordings were converted from their initial .webm format to .mp4 at a bitrate of 192 kbps from which the audio was extracted as a .wav file. All real audio files were converted to a .mp3 format with a sample rate of 44kHz, with an amplitude normalized between $-1$ and 1, and with silences at the start and end removed. In this study, we only used a subset of the audio clips, which had a mean duration of 5.37s (min: 0.88s, max: 52.57).

### Voice cloning

A voice clone of each of the 220 speakers was generated using the ElevenLabs’ *Instant Voice Cloning* API. For each speaker, a cloned voice model was first synthesized using the audio from just the first two prompts in the DeepSpeak dataset as input. Transcripts of speakers’ responses to other prompts were then used to create a cloned version of each original audio clip (32 total per speaker). For scripted responses, we assumed that the speaker correctly repeated the prompt; for unscripted responses, OpenAI’s *Whisper*^17^ was used to transcribe the audio. Cloned speaker audio files were converted to the same format, sample rate, and amplitude as the real speaker audios.

### Voice matching

For one of our studies, participants were presented with two real voices with different speaker identities. When people heard two speakers with different identities, we wanted the voices to be similar to each other. Thus, for each speaker in our dataset, we also determined another speaker with a perceptually similar voice. This voice matching was performed by first extracting a 192-D TitaNet embedding^18^ of the same scripted sample. The closest matching voice was determined by finding the voice of another speaker (with replacement) with the maximal cosine similarity between extracted embeddings (the mean similarity was 0.6 in the range $[-1,1]$). Audios were paired by scriptedness to ensure comparisons were made between similar contexts.

### Study design

We examined people’s perception of AI-powered voice clones in two perceptual studies.

**Fig. 1:**
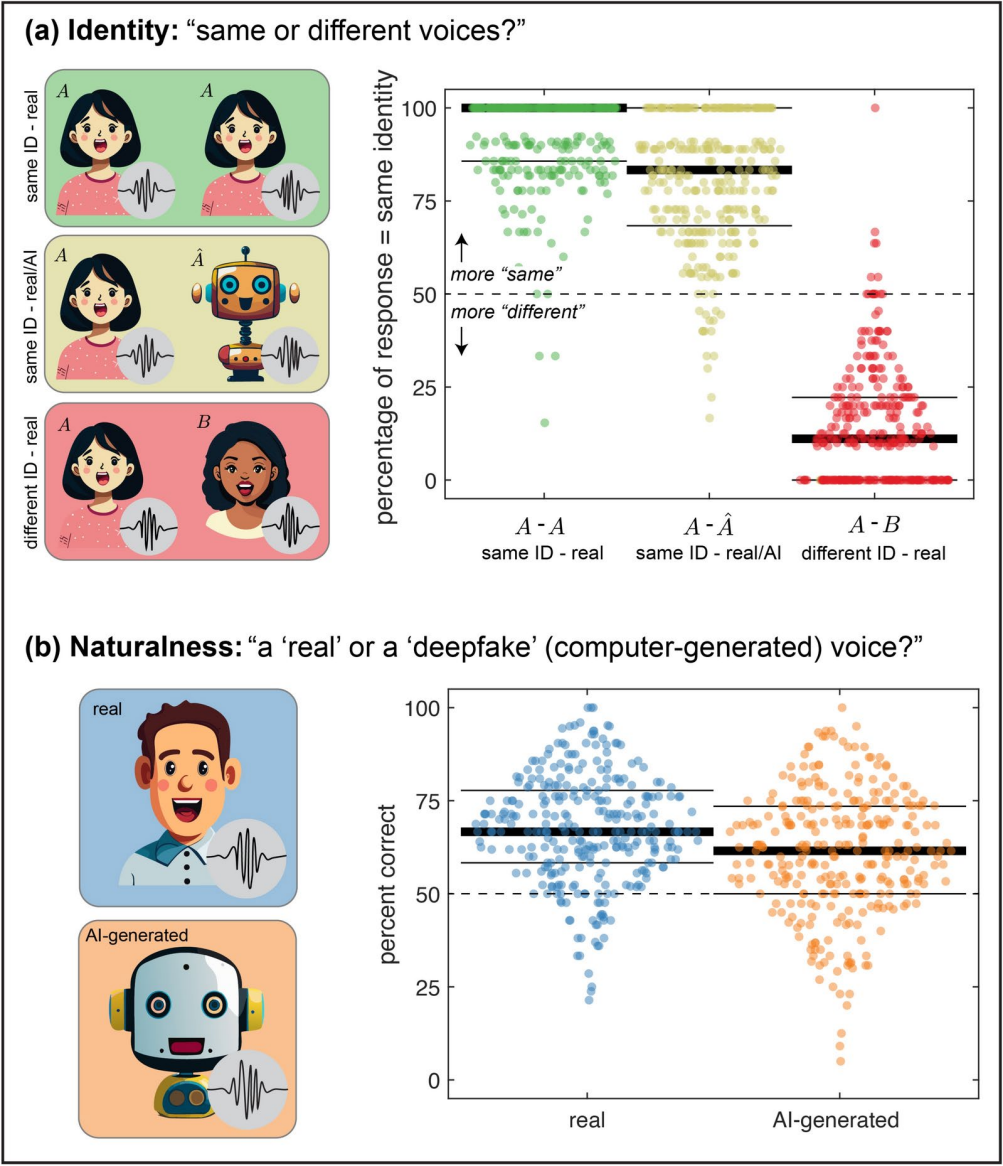
Study methods and results. (**a**) In the identity study, participants were presented with pairs of audio clips and asked if they were from the same or different people. We present results for three conditions: both voices were the same identity and a real person ($A-A$, green), both voices were the same identity but one clip was an AI-clone ($A-\hat{A}$, yellow), and the voices were from real but different identities ($A-B$). Results are plotted as the percentage of trials on which each participant responded “same identity.” Thick lines indicate medians and thin lines indicate $25\text{th}$ and $75\text{th}$ quantiles. The $50\text{th}$ – $25\text{th}$ – $75\text{th}$ quantile for the three remaining, unplotted conditions are as follows: [same identity] / [AI-clone speaker] ($\hat{A}-\hat{A}$) $100.0\%$ – $100.0\%$ – $100.0\%$, [different identity] / [AI-clone speaker] ($\hat{A}-\hat{B}$) $0.0\%$ – $0.0\%$ –$20.0\%$, and [different identity] / [real and AI-clone speaker] ($A-\hat{B}$) $3.6\%$ – $0.0\%$ – $16.7\%$. (**b**) In the naturalness study, participants were presented with audio clips one at a time and asked whether the voice was real or AI generated. Results are plotted in the same manner as panel (**a**), except now the ordinate indicates percent correct for real (blue) and AI-generated (orange) voices.

In the *identity* study (Fig. 1a, left), participants listened to two voices back-to-back (saying something different) and were asked to specify if the voices were from the same identity. Participants were randomly assigned to receive one of 10 batches comprising a randomized set of 44 stimuli (i.e., 44 unique random voice pairings). Thirty of these stimuli contained scripted single-sentence responses, 10 were unscripted responses, and 4 were attention checks (see below). There was no stimulus overlap between batches. Because the voice pairings were randomized, each stimulus could fall into six possible conditions. The three conditions of core interest for our analyses were:

- [same identity] / [real speaker] ($A-A$),
- [same identity] / [real and AI-clone speaker] ($A-\hat{A}$),
- [different identity] / [real speaker] ($A-B$).

There were also trials that fell into three additional conditions, which ensured that the structure of the study could not be learned by participants over time:

- [same identity] / [AI-clone speaker] ($\hat{A}-\hat{A}$),
- [different identity] / [AI-clone speaker] ($\hat{A}-\hat{B}$),
- [different identity] / [real and AI-clone speaker] ($A-\hat{B}$).

Participants were asked only to judge identity and were not told that some voices may be AI generated.

In the *naturalness* study, participants listened to one voice at a time and were asked to classify it as real or AI generated (Fig. 1b, left). The randomization and assignment of stimuli was identical to the identity study, except in this case each stimulus was one audio clip rather than two. Half of the audio clips were real and half were AI generated. Participants were not told of this distribution. For both studies, participants did not receive any explicit instructions to use headphones, earphones or speakers. The full instructions are included in the Supplementary Information.

### Listener participants

A total of 604 participants were recruited from the Prolific crowd-sourcing platform, split into two groups of 304 and 300 for the identity and naturalness studies. Listener ages ranged from $18-77$ years (mean=35, sd=12), with 293 male, 294 female, 11 non-binary and 6 not providing their gender. 411 listeners identified as White/Caucasian, 132 as Black/African American, 43 as Asian, 19 as American Indian/Alaska Native, 4 as Native Hawaiian/Other Pacific Islander, 30 as other, and 5 preferred not to share.

This study was approved by the UC Berkeley Committee for Protection of Human Subjects. Participants provided informed consent prior to participation and data collection was performed in accordance with relevant guidelines and regulations.

Before beginning the study, participants were given an overview of their task (either in terms of judging identity or naturalness) and tested their hardware. For the naturalness study, participants were given two examples of real voices and two examples of AI-generated voices to set expectations as to the realism of the voices.

To ensure that participants were paying attention, four attention checks were randomly distributed throughout. These checks consisted of audio clips that clearly described the correct answer to be selected. Participants who failed any of these attention checks were removed from subsequent analysis (the totals reported above only include participants who passed all attention checks). Participants could not respond without having listened to the entire audio(s). Participants were paid $5 for their time.

### Statistical analysis

For each participant in the *identity* study, we calculated the percentage of stimuli for which they responded that the identities were the same, separately for each stimulus condition (($A-A$), ($A-\hat{A}$), ($A-B$), etc). The distribution of responses across conditions deviated substantially from normality, so we adopted non-parametric statistics to examine differences between conditions. We used single sample signed rank tests (with test statistic W) to examine differences from chance ($50\%$) separately for each condition, then a Friedman test was used to examine whether there were significant differences between conditions. Pairwise follow up tests were conducted via paired sample rank sum tests.

For the *naturalness* study, the response distributions were approximately normally distributed, so we used single and paired sample t-tests to examine differences from chance ($50\%$) for stimuli that were real and AI generated, as well as to directly compare responses for these two stimulus types. We report effect size as Cohen’s D.

For all analyses, we adopted a significance threshold of $p < 0.05$ and applied Bonferroni correction when the same analysis was run on multiple conditions. Because six participants in the naturalness study did not provide their gender, the participant count for these analyses was reduced from 300 to 294.

Because we used a large dataset of naturalistic stimuli and designed our study to obtain a large listener participant sample, audio clips in this experiment varied in length and each participant only listened to a subset of stimuli (the randomized batches). To examine how these, and other variables, might affect performance, we therefore fit the trial-by-trial responses with mixed-effects logistic regression models.

For the *identity* study, we focused on fitting performance on trials with the same real identity ($A-A$) and those with the same identity but one AI-generated clone ($A-\hat{A}$) – that is, to keep the model relatively simple we only fitted the data from these two conditions. For the *naturalness* study, the model was fitted to all data. Each model also included random effects (intercepts) per participant and per stimulus batch. In addition to the stimulus condition, the fitted models included the following predictors: total audio duration (continuous), scripted vs unscripted (categorical), listener gender (categorical), listener age (continuous), speaker gender (categorical), speaker age (continuous). The combined audio duration of both audios was used.

For both models, the continuous predictors, age and duration, were scaled using Z-score normalization and robust scaling, respectively.

## Main results

### Identity

When participants listened to a pair of real audio clips with the same speaker ($A-A$), they were on average highly accurate. That is, they overwhelmingly indicated that it was the same person speaking in both clips: $50\text{th}$ – $25\text{th}$ – $75\text{th}$ quantile of $100.0\%$ – $85.7\%$ – $100.0\%$, as shown in Fig. 1a, green. Indeed, $58.6\%$ of participants indicated that it was the same identity on every trial for these stimuli. When one of the clips was an AI-clone ($A-\hat{A}$), participants also overwhelmingly judged that it was the same person speaking in both clips: $50\text{th}$ – $25\text{th}$ – $75\text{th}$ quantile of $83.3\%$ – $69.2\%$ – $100.0\%$ as shown in Fig. 1a, yellow. However, only $26.6\%$ of participants indicated the same identity on every trial. This finding suggests that AI-clones are highly, but not uniformly, convincing. It is also possible that participants were simply biased to indicate that all speaker pairs were the same. The data, however, show that this was not the case: when participants listened to two different, but similar, voice identities ($A-B$), they rarely indicated that the speakers had the same identity: $50\text{th}$ – $25\text{th}$ – $75\text{th}$ quantile of $11.1\%$ – $0.0\%$ – $22.2\%$, as shown in Fig. 1a, red.

Statistical tests indicate that responses in each of these conditions are significantly different from chance ($A-A$: W = 94.5, $p < 0.001$; $A-\hat{A}$: W = 693.5, $p < 0.001$; $A-B$: W = 301.5, $p < 0.001$). There are also significant differences between conditions (Friedman test = 517.0, $p <0.001$). Follow up pairwise tests indicate that each pair of conditions differ significantly ($A-A$ vs $A-\hat{A}$: W = 3605.5, $p < 0.001$; $A-A$ vs $A-B$: W = 0.0, $p < 0.001$; $A-\hat{A}$ vs $A-B$: W = 0.0, $p < 0.001$). Responses for the other three conditions are generally consistent with these results and are reported in the caption of Fig. 1a.

The responses to the $A-A$ and $A-\hat{A}$ conditions were further investigated with a logistic regression model (Table 1). As compared to the $A-A$ condition, the $A-\hat{A}$ condition is associated with significantly fewer “same” responses. There is also a significant effect of speaker gender: the voices of male and non-binary genders are more often judged “same” as compared to female speakers. Interestingly, both combined audio duration and scriptedness have significant effects: longer clips and scripted clips are associated with more frequent “same” responses. We will return to explore these effects in more detail in the Exploratory results.

### Naturalness

In the naturalness study, people were asked to directly judge whether individual audio clips were real or AI generated (irrespective of identity). This proved to be a more challenging task, Fig. 1b. When the audio clip contained a real voice, participants were correct on average $67.4\%$ of the time (standard deviation $14.8\%$). Similarly, when the audio clip was one of our AI clones, participants were correct $60.8\%$ of the time (standard deviation $16.7\%$). Indeed, $9.7\%$ and $21.0\%$ of participants were at or below chance for the real and AI-generated stimuli, respectively.

Nonetheless, for both stimulus types, average performance is significantly greater than chance with a medium or large effect size (real: t(299) = 20.4, $p <0.001$, D = 1.177; AI generated: t(299) = 11.2, $p < 0.001$, D = 0.648). A pairwise comparison between stimulus types indicates a small but statistical significant difference associated with stimulus type (t(299) = 4.6, $p < 0.001$, D = 0.263).

Consistent with these results, the logistic regression model (Table 2) indicates a significant effect of stimulus type (real vs AI generated) on accuracy. None of the listener or speaker demographics are associated with statistically significant effects. However, as in the identity study, there are, again, significant effects associated with audio duration and scriptedness. In this study, longer clips and unscripted clips are associated with more accurate judgments of naturalness.

**Table 1:** Generalized linear mixed-effects model for the identity study showing coefficients, z-values, *p*-values, and $95\%$ confidence intervals. The model includes fixed effects for predictor variables and random intercepts by group. Predictor variables include *combined duration* (continuous), *listener age* (continuous), *speaker average age* (continuous), as well as *scriptedness* (scripted vs. unscripted), *condition* (real vs. AI-clone), and *gender* (categorical), with grouping by ResponseId (participant) and batch_number.

Predictor	Coef.	z	*p*-value	95% CI Lower	95% CI Upper
Intercept	2.619	18.201	<0.001	2.337	2.901
Condition assigned ($A-\hat{A}$)	−0.988	−10.814	<0.001	−1.167	−0.809
Combined duration	0.533	3.953	<0.001	0.269	0.797
Scripted (vs Unscripted)	−1.331	−5.831	<0.001	−1.778	−0.883
Listener gender (Male)	−0.198	−1.594	0.111	−0.442	0.046
Listener gender (Non-Binary)	−0.476	−1.195	0.232	−1.258	0.305
Listener age	−0.079	−1.293	0.196	−0.199	0.041
Speaker gender (Male)	0.376	4.227	<0.001	0.202	0.551
Speaker gender (Non-Binary)	0.736	3.028	0.002	0.260	1.213
Speaker average age	−0.005	−0.133	0.894	−0.087	0.076
Random effects					
Group: ResponseId (304 levels)	Standard deviation = 0.745				
Group: Batch Number (10 levels)	Standard deviation = 0.257

**Table 2:** Mixed-effects logistic regression model for the naturalness study showing coefficients, z-values,* p*-values, and $95\%$ confidence intervals. The model includes fixed effects for predictor variables and random intercepts by group. Predictor variables include *duration* (continuous), *listener age* (continuous), *speaker age* (continuous), as well as *scriptedness* (scripted vs. unscripted), *condition* (real vs. fake), and *gender* (categorical), with grouping by ResponseId (participant) and batch_number.

Predictor	Coef.	z	*p*-value	95% CI Lower	95% CI Upper
Intercept	0.497	10.650	<0.001	0.405	0.588
Condition assigned (AI generated)	−0.229	−5.614	<0.001	−0.309	−0.149
Duration	0.160	6.640	<0.001	0.112	0.207
Scripted (vs Unscripted)	0.311	3.153	0.002	0.118	0.505
Listener gender (Male)	−0.011	−0.211	0.833	−0.110	0.089
Listener gender (Non-Binary)	0.274	1.233	0.218	−0.162	0.710
Listener age	−0.004	−0.162	0.871	−0.054	0.046
Speaker gender (Male)	0.020	0.493	0.622	−0.059	0.099
Speaker gender (Non-Binary)	−0.126	−0.866	0.387	−0.410	0.159
Speaker age	0.028	1.359	0.174	−0.012	0.067
Random effects					
Group: ResponseId (294 Levels)	Standard deviation = 0.263				
Group: Batch number (10 Levels)	Standard deviation = <0.001				

Together with the results of the identity study, these results reflect a status quo whereby people can often be tricked into thinking that AI-generated clones have the same identity as a real speaker, and cannot reliably detect when a voice they hear is AI generated.

## Exploratory results

AI-powered phone scams can range from brief, scripted robocalls to fully-fledged conversations. Since our analyses revealed a significant effect of audio clip length and scriptedness on performance in both studies, we conducted an exploratory follow up to understand better how these factors affect performance. We focused on the naturalness task and aimed to investigate whether the effect of clip length on performance in the naturalness study was due, at least in part, to the fact that the unscripted responses tended to be longer (i.e., was improved performance caused by clip length or unscripted content?). This study contained a new set of 25 additional audio clips with longer scripted and shorter unscripted audio clips. It also included combined scripted-unscripted clips, for example, a speaker reading aloud a written question and then answering it. These audio clips were played to 30 new participants.

We first conducted exploratory analyses separately on clip length and scriptedness for this new dataset. For the clip length analysis, we computed the average accuracy across all participants for each clip. This analysis revealed a weak to moderate (but statistically significant) positive relationship between audio duration and the accuracy of identifying the audio as real or AI generated (Spearman $r_s$ = 0.245, $p < 0.001$). Scriptedness was associated with a statistically significant difference in performance in this study (Friedman test = 13.0, p = 0.001), and additionally, we observed a qualitative trend consistent with the main study, whereby the median accuracy was highest for combined audios ($83.3\%$), followed by unscripted audios ($76.7\%$) and scripted audios ($56.7\%$).

A logistic regression that included effects for both scriptedness and audio duration indicated that these effects were not statistically significant, likely because clip length and scriptedness were still somewhat correlated even in this dataset. However, the coefficients were consistent with the findings of the logistic regression model from the naturalness study, which identified positive relationships for both duration and scripting. Therefore, these results provisionally support the conclusion that both the duration of an audio and its scriptedness influence accuracy, but a larger dataset with a wider variety of audio clips would be necessary to confirm this.

These followup results, in conjunction with the findings from the naturalness study, suggest a potential strategy for listeners to better identify fraudulent AI-voices: engage the speaker in a longer conversation by, for example, asking open-ended questions.

## Discussion

Here, we discuss the implications of these results further and report an exploratory analysis of participant strategies.

### Self-reported listener strategies

**Fig. 2:**
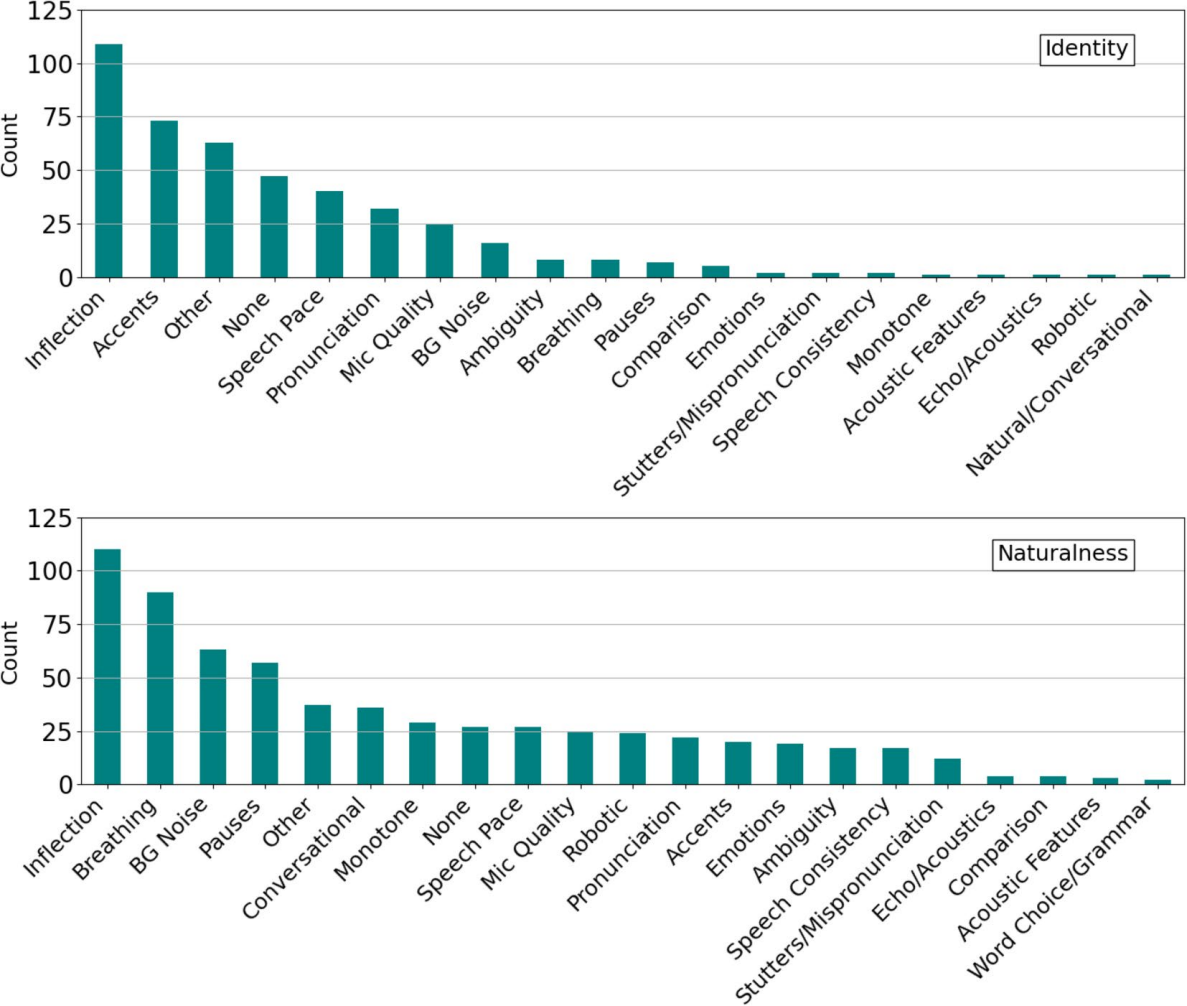
The 21 most frequent thematic codes as reported by participants in the naturalness (top) and identity (bottom) studies. See also Fig. 3.

At the end of each study, participants were asked to share any tactics they used to differentiate between same/different voices (identity study) and real/AI-generated voices (naturalness study). Keywords were extracted from their responses using a qualitative coding analysis and grouped into thematic codes. As shown in Fig. 2, the top three most frequent codes in the identity study were “inflection,” “accents,” and “other.” For the naturalness study, these were “inflection,” “breathing,” and “background noise.”

We also found evidence that these strategies were impacting performance by looking at participant responses. Specifically, six audio recordings were correctly classified by all participants in the naturalness study, all of which were real, and five of which were unscripted. Qualitatively, we observed that these six recordings did indeed contain audible background noise, opening mouth clicks, and several disfluencies (um, ah, etc.). At the same time, these cues were not necessarily diagnostic in general. For example, “background noise” was mentioned 79 times across both studies, yet, upon analyzing the average background noise in both datasets, no significant effects on performance were found. Indeed, given the variety of background noise conditions that can occur on phone calls, it seems unlikely that this cue holds a key to detecting AI voice clones on the phone. We therefore suggest that a good listener strategy would be to disregard background noise as a cue and focus on the qualities of the caller’s voice.

**Fig. 3:**
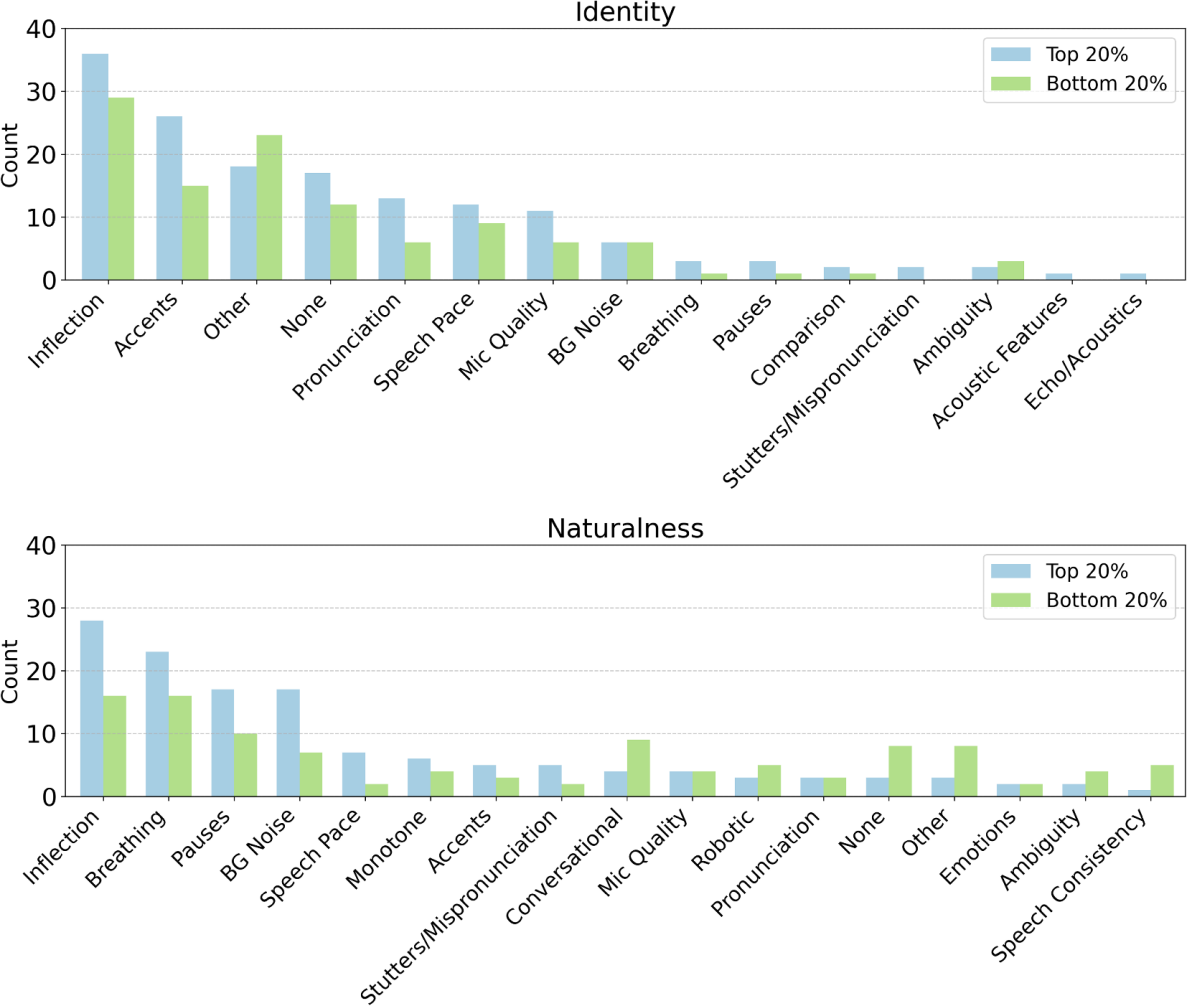
The most frequent thematic codes as reported by the top and bottom $20\%$ performing participants for the naturalness (top) and identity (bottom) studies. See also Fig. 2.

Additionally, we examined the strategies of the top and bottom $20\%$ of performers for each study, Fig. 3. Interestingly, for the identity study, “inflection” remained the most popular strategy for both top and bottom performers, but “accents” were more popular with top performers than bottom. For the naturalness study, the top performers also relied upon “inflection” as their most popular strategy, while the bottom performers relied equally upon “inflection” and “breathing.” While both groups relied upon “pauses” and “background noise” as popular strategies, top performers relied more frequently upon “speech pace,” while bottom performers tended to favor “conversational” strategies. These findings suggest, while some authenticity cues such as breaths may be misleading, speech pace and accent may still offer effective indicators for detecting AI voice clones.

### Gender differences

Although there were no effects of gender (or other demographics) in the naturalness study, we found an effect of gender in the identity study. In particular, the voices of male and non-binary speakers were associated with more “same” responses as compared to female speakers. This could be the result of a bias in the AI training in which female voices are underrepresented and so AI-generated male voices are more identity preserving. However, we were also curious whether this finding might reflect an additional perceptual effect related to gender. For example, the well-known cross-race effect describes the phenomena in which people more easily recognize faces that belong to our own racial group, or the group to which we have most exposure^19^. We wondered if there was a similar cross-gender effect in voice perception.

To investigate this possibility, the main logistic regression models described in Tables 1 and 2 were modified to replace the speaker and listener gender predictors with a single same/different gender predictor corresponding to whether the listener and speaker were of the same gender (because non-binary listeners and speakers were heavily underrepresented in our dataset, they were excluded from this analysis). For both studies, we found no evidence for a cross-gender effect. That is, participants were not notably better or worse at detecting AI-generated voices within or across their own gender.

### Forensic techniques

While modern forensic techniques^20^ are better than humans at distinguishing between certain characteristics of real and AI-generated voices, these techniques typically operate asynchronously, making it difficult to protect consumers on phone/video calls. While synchronous techniques can operate at the source of a call, this raises serious privacy concerns that would need to be addressed. Any such technology would also have to have exceedingly high accuracy to avoid false alarms and giving a false sense of security against AI-powered voice scams.

Our reporting of people’s ability to detect AI-generated voices derives from a task in which their attention is fully drawn to either the identity or realism of a voice. In real-world scenarios, people may be less attentive to the voices and therefore more likely to be fooled.

Another intervention that may prove useful (albeit not perfect) in mitigating the risk of AI-powered scams is the insertion of difficult to remove and easy to identify, imperceptible watermarks into AI-generated voices. With the appropriate software at the receiver, AI-generated voices can be easily identified. If, however, all AI-powered voice generators do not deploy this technology, scammers would simply migrate to those services that opt out of this security protocol.

## Conclusions

The quality and realism of AI-generated media is rapidly improving. Given the results reported here, there is good reason to believe that AI-generated voices will soon be indistinguishable from real ones both in terms of naturalness and identity. While this should be considered a triumph for those on the generative side, it raises real concerns for public safety. Our results highlight that relying on human perception to detect AI-generated voice clones is no longer consistently reliable. Thus, improved technologies that can detect AI-generated voices while protecting the user’s privacy will become essential tools for preventing phone-based – and eventually, video-based – frauds.

## Data Availability

Audio data available at https://huggingface.co/datasets/faridlab/deepspeak_v1. Anonymized speaker and listener data available at https://doi.org/10.5281/zenodo.13654688.
